# Inflammatory bowel disease causing retroperitoneal varicosity mimicking a renal artery aneurysm: A novel case report and literature review^☆^^☆^

**DOI:** 10.1016/j.radcr.2022.01.003

**Published:** 2022-01-18

**Authors:** Areez Shafqat, Shameel Shafqat, Belal Nedal Sabbah, Abdullah Shaik, Wael Khalil Alfehaid, Syed Shafqat Ul Islam

**Affiliations:** a College of Medicine, Alfaisal University, Riyadh, Kingdom of Saudi Arabia; b Medical College, Aga Khan University, Karachi, Pakistan; c Department of Radiology, King Salman Hospital, Riyadh, Kingdom of Saudi Arabia

**Keywords:** Inflammatory bowel disease, Retroperitoneal mass, Varicosity, Retroperitoneal surgery, Abdominal imaging

## Abstract

A 17-year-old female presented to our hospital complaining of bloody diarrhea 4-6 times per day for the past month. She was a known case of inflammatory bowel disease noncompliant to her medications. Abdominal computed tomography revealed an unusually dilated mass in the retroperitoneum at L2 vertebral level connecting the lumbar and left renal veins. The renal artery was visualized separately, and a diagnosis of communicating vein varicosity was made. This lesion can be misleading on imaging, hence our aim to disseminate our findings to practicing radiologists. The differential diagnosis of these lesions include retroperitoneal lymphadenopathy, renal artery aneurysms, and testicular cancers causing retroperitoneal lymphadenopathy. To our knowledge, this is the first case to be reported in association with inflammatory bowel disease, perhaps providing a novel insight into the pathogenesis of this lesion that has not been considered in the contemporary literature.

## Introduction

Inflammatory bowel disease (IBD) is characterized by an idiopathic chronic inflammatory state of the gastrointestinal (GI) tract and encompasses 2 distinct disease states: ulcerative colitis (UC) and Crohn's disease (CD). The clinical presentation of UC and CD overlap, the major difference between the 2 being the lack of small bowel involvement in UC [1].

Numerous studies have confirmed the elevated risk of venous thromboembolism (VTE) in IBD patients. The risk of thrombosis in IBD patients is 1.7 times higher compared to controls [2], the most common site for thrombosis in IBD is mesenteric vein thrombosis and portal vein thrombosis [2], and management guidelines accordingly recommend VTE prophylaxis in IBD patients who were hospitalized after a disease flare-up [3]. Collectively, VTE phenomena are important findings in IBD that significantly affect patient prognosis and treatment [4], [5], [6].

Here, we describe the radiologic detection of varicosity in the communicating vein between the left renal vein (LRV) and left ascending lumbar vein (LALV) in an inflammatory bowel disease patient, a novel association with IBD. This finding is important both radiologically and surgically, and we hope these specialties themselves with this finding.

## Case report

An 17-year-old female, known case of IBD, presented with the chief complaint of bloody diarrhea 4-6 times per day for the past month. Oral steroids and 5-aminosalicylic acid had previously been prescribed for the UC, and the patient was asymptomatic until independently discontinuing both medications, which caused symptoms to flare up.

**Fig. 1:**
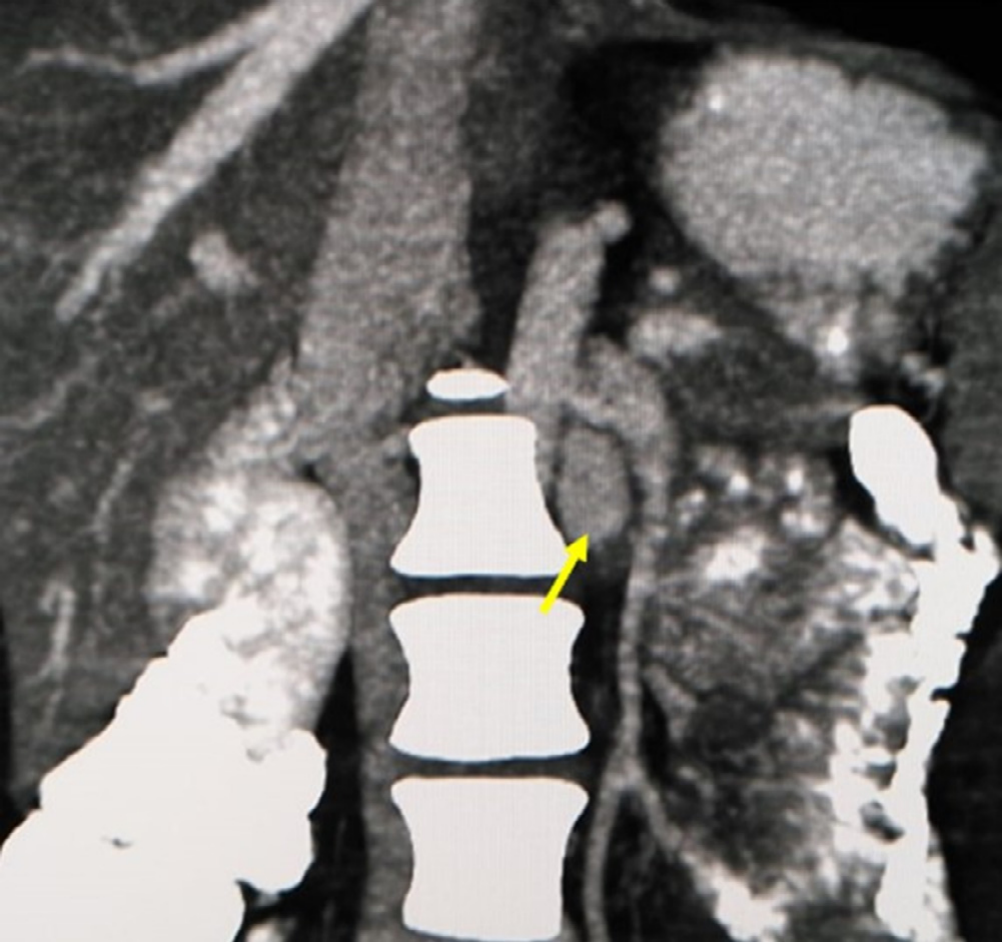
Coronal reconstructed MIP image (venous phase study) shows a dilated vascular channel in the left paravertebral region at L2 level (arrow).

**Fig. 2:**
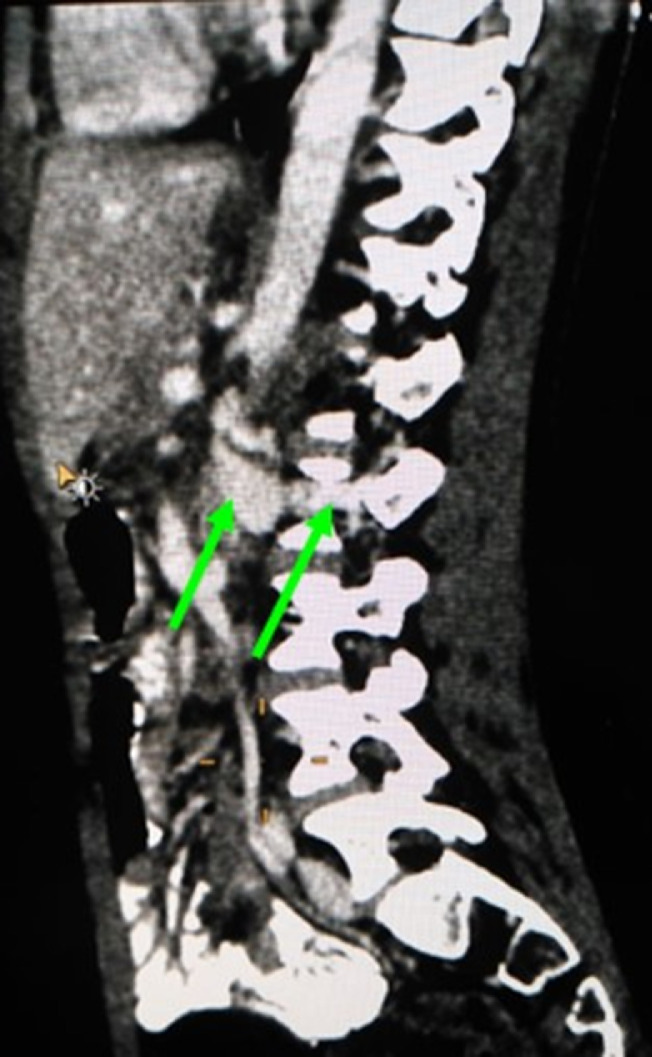

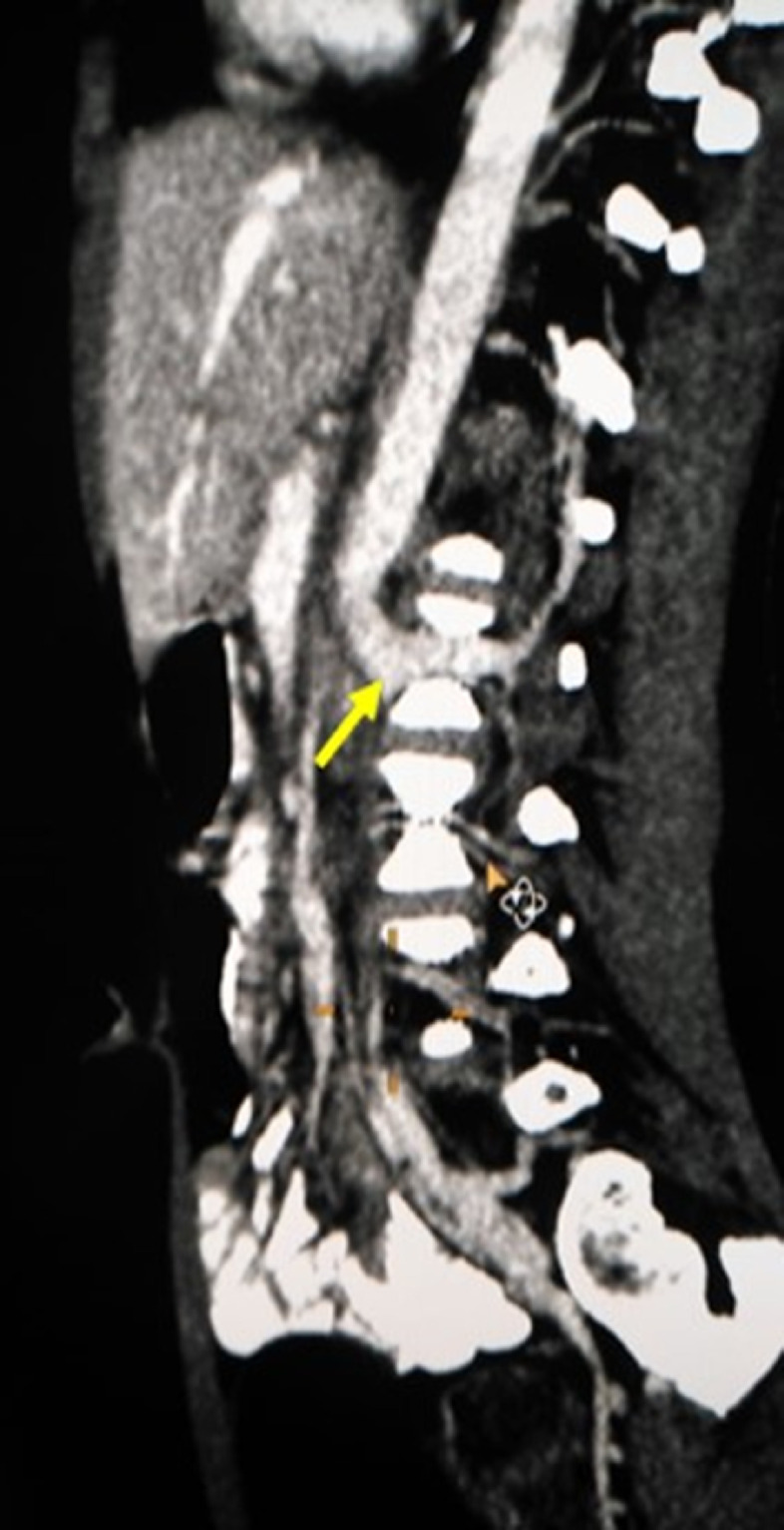
Sagittal reconstructed MIP images (venous phase study) reveal left paravertebral vascular dilatation (yellow arrow) which is connected to the vertebral venous plexus (green arrow).

**Fig. 3:**
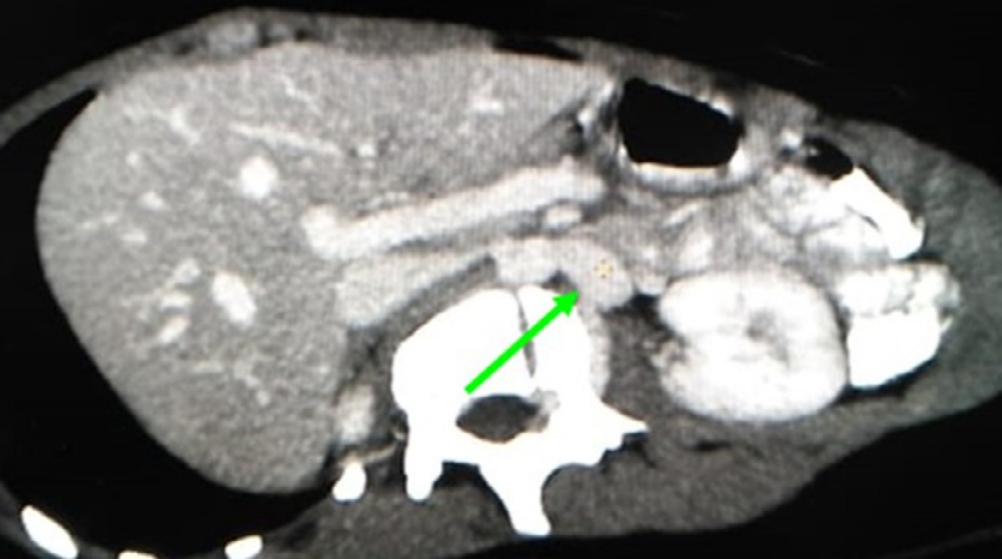
Axial oblique reconstructed MIP image (venous phase study) shows dilated left ascending paravertebral vein draining into the left renal vein (arrow).

She was referred for radiologic assessment of her IBD. However, contrast-enhanced computed tomography (CT) scan of abdomen and pelvis also revealed an unusual aneurysmal dilatation of a vascular channel, measuring 13 × 15 × 15 in the transverse, anteroposterior, and craniocaudal dimensions, respectively, in the left paravertebral gutter at L2 vertebral level (Fig. 1). On sagittal and axial reconstructions, the channel communicated medially with the lumbar veins and vertebral venous plexus and superiorly with the left renal vein (Fig. 2, Fig. 3). The renal artery was visualized separately with normal caliber. No underlying structural or vascular abnormalities of the liver, gallbladder, spleen, kidneys, and suprarenal glands were observed. Lastly, no para-aortic lymphadenopathy was seen. On this basis, a diagnosis of varicosity in the left ascending lumbar communicant vein mimicking a renal artery aneurysm was reached.

We continued our patient on conservative management with 5-aminosalicylic acid for the IBD and advised her for regular follow-ups.

## Discussion

The left ascending lumbar vein (LALV) courses between the psoas major muscle and lumbar vertebrae, connecting the common iliac vein, 4 lumbar veins, vertebral venous plexus, and iliolumbar veins before draining into the azygos/hemiazygos venous system. The LALV has also been found to communicate with the left renal vein (LRV) with an estimated prevalence of 30%-46.4% on cadaver studies [7]. Another study revealed this venous communication in 35 out of 100 (35%) randomly chosen patients [8]. Collectively, these results implicate an anastomosis between the LRV and LALV as being quite frequent. However, varicosities in these communicating veins are very rarely reported; we found only 4 reported cases in the literature. This also may be an indication of LALV-LRV communicating vein varicosities being frequently overlooked, hence our aim to disseminate these findings among radiologists.

Although rare, this finding is of utmost importance to surgeons performing renal, aortoiliac, and other retroperitoneal surgeries, as unawareness could lead to accidental injury and life-threatening retroperitoneal hemorrhage [9,10]. This makes accurate radiologic diagnosis paramount. However, LALV communicant vein varicosities can be misleading in imaging studies as they mimic retroperitoneal lymphadenopathies, adrenal masses, or renal artery aneurysms [11], [12], [13]. A CT scan and phlebographic studies constitute essential investigations to reveal the vascular nature of this entity [12]. Visualization of the renal artery as separate excludes a renal artery aneurysm. In short, familiarity with this clinical entity is necessary for radiologists to facilitate a correct imaging diagnosis and avoid potentially severe surgical complications.

The first reported case was a 34-year-old man who underwent orchiectomy for testicular cancer, making para-aortic lymphadenopathy a pitfall in diagnosis since testicular carcinomas exhibit lymphatic spread to the para-aortic nodes [12]. Three case reports have been published since, with misdiagnosis as retroperitoneal lymphadenopathy and renal artery aneurysms a prevailing theme [13], [14], [15]. However, none of the cases could provide an etiology. Interestingly, this clinical entity usually manifests in young adult females. Whether this association is simply down to chance or another reason, is unknown. Nevertheless, this association prompted us to assess for potential pelvic vascular congestion, but to no avail.

Since varicosities, by definition, evolve secondary to valve incompetency or increased backflow following obstruction, potential causes in our case might include pathologies such as thrombi within the LRV or inferior vena cava, leading to valvular incompetency and consequent retrograde flow into the communicating vein. The nutcracker syndrome, characterized by compression of the LRV between the superior mesenteric artery and abdominal aorta, obstructs the LRV. These more untoward etiologies must be excluded when evaluating such lesions. In the case presented here, the LALV-LRV varicosity might have developed as a consequence of IBD, but these postulations are purely theoretical. Theoretically, IBD, like other chronic inflammatory conditions, raises the possibility of systemic thrombotic events by precipitating a hypercoagulable state. Indeed, IBD is an independent risk factor for venous thromboembolic events [16]. Alternatively, this could perhaps broadly implicate LALV-LRV communicant vein varicosities as consequences of inflammatory conditions predisposing to thrombotic events.

No objective direction currently exists on the management of this finding. Since these varices are predominantly asymptomatic, a conservative approach is recommended, with definitive treatment aimed at resolving the underlying cause – likely IBD in this case. With the aforementioned hypothesis that LALV-LRV varicosities are a manifestation of underlying inflammatory states, clinical and radiologic evaluation for such conditions should include these etiologies. Also, treating the inflammatory state may constitute a valid therapeutic strategy.

## Conclusion

We reported varicosity in the LRV-LALV communicating vein in an IBD patient, which from our literature search has not been reported previously. We encourage practicing radiologists to acquaint themselves with the imaging approach to these lesions to prevent the potentially disastrous complications of misdiagnosis. The differential diagnosis of these lesions includes testicular carcinomas, renal artery aneurysms, and other retroperitoneal mass lesions, such as lymphadenopathy. All previous cases reporting an LRV-LALV communicant vein varicosity suggested an idiopathic etiology, but our case may provide a clue to the underlying pathogenesis of this finding.
